# Acknowledgement of manuscript reviewers 2015

**DOI:** 10.1186/s13031-016-0072-y

**Published:** 2016-02-22

**Authors:** Ruwan Ratnayake, Bayard Roberts, Michelle Gaffey, Adrianna Murphy

**Affiliations:** International Rescue Committee, New York City, USA; London School of Hygiene and Tropical Medicine, London, UK; Centre for Global Child Health, Toronto, Canada

## Abstract

The editors of *Conflict and Health* would like to thank all our reviewers who have contributed to the journal in Volume 9 (2015).

**Adam Coutts**

UK

**Alex Cohen**

UK

**Bayard Roberts**

UK

**Olivier Degomme**

Belgium

**Debby Guha-Sapir**

Belgium

**Claude De Ville De Goyet**

Belgium

**Barbara Tomczyk**

USA

**Jason Nickerson**

Canada

**Michelle Gaffey**

Canada

**Katherine Muldoon**

Canada

**Ann Montgomery**

Canada

**Nadia Akseer**

Canada

**Chen Reis**

USA

**Chesmal Siriwardhana**

Sri Lanka

**Claudia Catani**

Germany

**Erin Wheeler**

USA

**Charlotte Hanlon**

Ethiopia

**Augusto Llosa**

France

**Richard Gosselin**

USA

**Verena Mauch**

Germany

**Walt Gill**

UK

**Grace Ryan**

UK

**Hedwig Deconinck**

USA

**Krishnamachari Srinivasan**

India

**Jeroen Ensink**

UK

**Jon Pedersen**

Norway

**Karl Blanchet**

UK

**Jocelyn Dejong**

Lebanon

**Lisa Hansen**

Canada

**Maartje Van Den Berg**

USA

**Marta Valenciano**

Spain

**Maureen Seguin**

UK

**Petros Isaakidis**

USA

**Sudesh Raj Sharma**

Nepal

**Maartje Hoetjes**

Netherlands

**Sumit Kane**

Netherlands

**Annick Lenglet**

Netherlands

**Oleg Bilukha**

USA

**Olivier Dagnelie**

Belgium

**Pablo Perel**

UK

**Preeti Patel**

UK

**Richard Garfield**

USA

**Ruwan Ratnayake**

USA

**Sara Casey**

USA

**Amira Shaheen**

Palestine

**Harry Shannon**

Canada

**Sian White**

UK

**Frederick Burkle**

USA

**Nathan Ford**

South Africa

**Martin Mengel**

Spain

**Triantafyllos Pliakas**

UK

**Grace Akello**

Uganda

**Katie Greenland**

UK

**Foyeke Tolani**

UK

**Egbert Sondorp**

UK

**Balsam Ahmad**

UK

**Peter Phillimore**

UK

**Rebecca Horn**

UK

**Mazeda Hossain**

UK

**Christopher Smith**

UK

**Patrick Bogue**

UK

**Joseph Fitchett**

UK

**Edward Roome**

UK

**Mahveen Siddiqui**

UK

**Margaret Kruk**

USA

**Ron Waldman**

USA

**Hannah Tappis**

USA

**Diane Morof**

USA

**Monica Onyango**

USA

**Peter Hovmand**

USA

**Barbara Lopes Cardozo**

USA

**Sarah Murray**

USA

**Rashed Shah**

USA

**Valerie Percival**

USA

**Lindsay Stark**

USA

**Stephanie Kayden**

USA

**Asha George**

USA

**Annie Sparrow**

USA

**Alessandro Demaio**

USA

**Gregg Greenough**

USA

**Nancy Glass**

USA

**Alexander Vu**

USA

